# Supplementary material for: SpottedPy quantifies relationships between spatial transcriptomic hotspots and uncovers environmental cues of epithelial-mesenchymal plasticity in breast cancer
Source: Genome Biol. 2024 Nov 11;25:289. doi: 10.1186/s13059-024-03428-y (PMC11552145; doi:10.1186/s13059-024-03428-y)
Supplement: Supplementary file 2 — Additional file 2: Review history. [file 13059_2024_3428_MOESM2_ESM.docx]

Review History

**First round of review**

**Reviewer 1**

**Were you able to assess all statistics in the manuscript, including the appropriateness of statistical tests used?**

**Yes:** Yes, they are suitable.

**Were you able to directly test the methods?**

No

**Comments to author:**

Eloise Withnell and colleague developed a python package SpottedPy to identify tumor hotspots and map spatial interactions within the cancer ecosystem. They used SpottedPy to examine EMT characteristics in breast cancer and revealed high EMT niches associated with angiogenesis and hypoxia, and shielded by myCAFs, macrophages and perivascular cell populations. Overall, the Spottedby can identify hotspots with interested function and dissect the neighborhood enrichment. The authors applied Getis-Ord G* statistic to find hotspots for functions and the infiltration of cell types, and focused on applying Spottedby to analyze EMT hotspots, but lacked assessment of the reliability of the hotspots they found, and did not conduct benchmarking of different feature-based spatial clustering, although they are mentioned in their future work in the discussion. In addition, SpottedPy can find multiple hotspots. The hotspots may be far apart in spatial location, and the features around them are also different. The author needs to add functions to help readers distinguish different hotspots.

Other comments are as follows:

1. Is there any inconsistency in EMT signature score distribution among different samples? The author only shows individual slides in each figure.
2. Hotspots of EMT, hypoxia, and angiogenesis may be co-located in the same spots. However, the authors show in the rightmost panel of Figure 2A that spots with these three characteristics are mutually exclusive.
3. The authors showed proliferation hotspots are closer to EMT coldspots, but the authors didn't show the distribution of proliferation hotspots.
4. What do the colors in Figure 2b and e mean?
5. How did the authors calculate the tumor perimeter? Cottrazm (10.1038/s41467-023-36560-7) developed by Xun et al. can delineate the location of tumor boundaries. The authors can compare with their method to confime whether similar results can be obtained.
6. In Figure 3e and f, macrophages ranked in the top 6 and top 2 respectively in the ranking close to EMT hotspots. What causes this difference?
7. Although macrophage and myCAF are relatively close to EMT hotspots, they are close to EMT hotspots at different spatial locations in Fig. 3. Do different EMT hotspots have different characteristics?
8. Three methods were used to compare the association of hotspot in EMT with other cell features, and whether the authors can compare the accuracy of these methods and whether these methods are applicable in other cancer types.
9. The authors only showed the distance of different cell types with EMT hotspots and coldspots, do different EMT characteristics of tumors affect their interactions with other cell types?
10. Hotspots and coldspots were identified based on Getis-Ord G* statistic. However, it seems that the spatial similarity between hotspot and other immune features in EMT is assessed by distance, and the description of coldspot seems to be less.
11. There are dozens of feature genes for immune suppression and ICB response. What are the author's criteria for selecting these genes?
12. The author needs to elaborate on how to use Spottedby. The methods need to be reorganized to make them clear to the reader.

**Reviewer 2**

**Were you able to assess all statistics in the manuscript, including the appropriateness of statistical tests used?**

No

**Were you able to directly test the methods?**

Yes

**Comments to author:**

Withnell et al. developed SpottedPy to identify tumor hotspots in spatial transcriptomics slides and explore their interaction with the tumor microenvironment (TME) at different scales. Using breast cancer as a model, the authors investigate epithelial-mesenchymal plasticity and emphasize locally stable niches linked to angiogenic and hypoxic regions. Additionally, the authors modified various parameters, such as neighborhood size and distance metrics, to assess the robustness and efficacy of SpottedPy. However, some conclusions in the paper still necessitate further clarification.

1.All conclusions are based on breast cancer, yet the paper's title does not reflect this. The authors should include more types of cancer to determine whether the conclusions regarding EMT are applicable across different cancer types

2.Which spots were used to calculate different types of hotspots? For example, in Figure 2A, the authors initially used STRACH to identify tumor spots and then applied Getis-Ord G* statistics to determine EMT hotspots or coldspots among them. However, were all spots used for hypoxic and angiogenic spots?

3.In Fig. 2A (right panel) and Fig. 3D (left panel), the authors overlaid different types of hotspots/coldspots on the same slide. An issue arises when different spot types overlap—how to assign names to these spots. For instance, there might be overlap between hotspot points for myCAF and hypoxic spots. How should this be addressed during the calculation process?

4.The author did not clearly explain how to calculate the tumor perimeter in the methods section. For example, some spots on the left side of Figure 2D are labeled as tumor perimeter, yet they are not surrounded by any tumor spots, which is evidently unreasonable. Therefore, the author should provide a rigorous mathematical formula for definition.

5.To some extent, using the shortest distance may not be suitable for calculating the similarity between two sets of spots. For instance, when EMT hotspots and proliferative hotspots share at least one common spot, their distance is zero. However, centroid-to-centroid distance also has significant limitations. For example, when EMT hotspots form irregular shapes or consist of multiple clusters distributed across different areas, the centroid approach may lead to erroneous conclusions.

6.Line 283-284: "We found that NK cells were the only cells consistently adjacent to EMT coldspots at various hotspot sizes." Based on Figure 2SA, it appears that this conclusion cannot be drawn

7.Sensitivity analysis should be emphasized. From Figure 4, it is evident that EMT hotspots vary significantly with different neighbor sizes, indicating instability. This raises questions about the meaningfulness of the hotspot definition.

8.All subsequent results depend on the accuracy of tumor spot identification. I believe that in sensitivity analysis, the authors should consider the impact of SCRACH on different parameters (e.g. setting K=?? clones), or rely on experts to assess the accuracy of tumor spot identification. This would make downstream analysis more practically meaningful.

9.What is the meaning of "#Rings around spot" in Fig. 5b? Based on Fig. 1A, the first ring should include 6 spots, why did the author specifically choose numbers like 1, 2, 3, 4, 5, 10, 20, 50 and 100?

10.What's the meaning of "number of components" in Fig.4d

11.What's the meaning of "correlation change" in Fig.5c.

**Authors Response**

**Point-by-point responses to the reviewers’ comments:**

**Summary of manuscript changes – Withnell and Secrier**

We are very grateful for the Reviewers’ suggestions, which have helped us to greatly strengthen and improve our findings. Here is a brief summary of the key modifications we have made to the analysis and manuscript following these suggestions:

1. We have improved our method of detecting EMT in the spatial transcriptomic spots by scoring the tumour epithelial cells identified in the scRNA-seq breast cancer reference set prior to deconvolution and using the deconvolved epithelial and mesenchymal tumour labels for downstream analysis. Whilst the trends with CAFs and macrophages remain the same, there were minor deviances in spatial relationship amongst other cell types. This has been adjusted in the results sections.
2. We have updated the cell subtype labels to be more granular in the deconvolution analysis, and updated the figures where appropriate to include the new cell type labels.
3. We have included additional functionality in SpottedPy to visualise and inspect individual EMT hotspots, and analyse the relationships at an individual hotspot level.
4. We clarify the distance metrics employed by SpottedPy and their advantages.
5. We have improved the statistical analysis across the slides, allowing the user to select mean, minimum or median value from each hotspot and use generalised estimating equations for repeated measures to more accurately determine the statistical significance of relationships across multiple hotspots across multiple slides.
6. We use simulations to demonstrate the distance metrics employed reflect biological expectations.
7. We have assessed the stability and robustness of hotspots and their relationship with other hotspots by adding gaussian noise and spot shuffling and measuring the downstream effects.
8. We have included an entire new section where we investigate intra- and intertumour heterogeneity of EMT hotspots and their interplay with the tumour microenvironment.
9. We have expanded our analyses to three other cancer types (basal cell carcinoma, pancreatic ductal adenocarcinoma and colorectal cancer) and include an entire new results section on this.
10. We have provided further explanations on the functionality and use of SpottedPy in the methods and at the GitHub repository.

A detailed, point-by-point description of the changes implemented to address the Reviewers’ comments is included in the next pages. The resubmitted manuscript contains highlighted changes in yellow (as tracking the changes became unwieldy), with amended Figures 1-6 and Supplementary Figures 1-7. Figures 4, 5e-f and Supplementary Figures 1, 2b,d-e, 3c-d, 4 contain entirely new analyses.

**Point-by-point response to reviewer comments**

**Reviewer #1**

Eloise Withnell and colleague developed a python package SpottedPy to identify tumor hotspots and map spatial interactions within the cancer ecosystem. They used SpottedPy to examine EMT characteristics in breast cancer and revealed high EMT niches associated with angiogenesis and hypoxia, and shielded by myCAFs, macrophages and perivascular cell populations. Overall, the Spottedby can identify hotspots with interested function and dissect the neighborhood enrichment. The authors applied Getis-Ord G* statistic to find hotspots for functions and the infiltration of cell types, and focused on applying Spottedby to analyze EMT hotspots, but lacked assessment of the reliability of the hotspots they found, and did not conduct benchmarking of different feature-based spatial clustering, although they are mentioned in their future work in the discussion.

We thank the Reviewer for the very useful comments and suggestions provided. We have run further analysis to assess the **reliability of hotspots**. We have addresses this in the Results sections, lines 348-356:

“To evaluate the stability of the spatial relationships, we introduced gaussian noise and spot reshuffling into the dataset and examined the persistence of these relationships (see Methods). This approach demonstrated that the method is robust to low levels of noise, but also effectively discriminates between biologically meaningful signals from those arising from random spatial distributions **(Fig. 5e-f).** While random noise simulated through spot reshuffling can mimic some aspects of structured data (**Supplementary Fig. 3c**), the hotspots are significantly smaller than those that are biologically relevant (**Supplementary Fig. 3d**). Crucially, the loss of specific associations among particular cell types when noise is introduced **(Fig 5e-f)** contributes to the reduction of false positives even if hotspots are identified(66). ”

With respect to the **benchmarking point**, we had indeed originally suggested in the discussion that it may be beneficial in the future to compare the results from the implemented Getis-Ord Gi* statistic to those that could be obtained using other approaches such as Kernel Density Estimation clustering (KDE). Following the Reviewer’s suggestion, we have had a closer look at the KDE method, and have realised that it estimates a density function and therefore does not return discrete clusters. Thus, it does not appear to be a comparable approach to the one implemented here. In fact, no existing methods perform both continuous signature spatial clustering and differential spatial relationship comparisons like SpottedPy does. Thus, to the best of our knowledge there are no direct comparisons available for this methodology. We have removed the reference to benchmarking against other feature-based clustering methods in the discussion, and further clarify the lack of other similar methods that it can be compared against at the present time in line 578-581:

“To the best of our knowledge there are no direct comparisons available for our SpottedPy methodology due to the unique nature of focusing on discrete spatial clusters of user-defined continuous signatures at expanding scales, and performing differential spatial relationships compared to a reference for downstream analysis. “

In addition, SpottedPy can find multiple hotspots. The hotspots may be far apart in spatial location, and the features around them are also different. The author needs to add functions to help readers distinguish different hotspots.

We thank the Reviewer for this excellent suggestion. We have now added functionality into SpottedPy that helps users identify and explore different hotspots, see new Figure 4 (especially panels c, d and e). We have addressed this in the Results, lines 312-326:

“This is further illustrated through visual inspection of the individual EMT hotspots and comparison with the LAM2+ APOE+ macrophage hotspots, enabled through SpottedPy functions **(Fig. 4b). […**] SpottedPy offers functionality to inspect the distance distributions for hotspots within a slide **(Fig. 4c),** and across each slide **(Fig. 4d).** Visualising these distributions highlights that whilst LAM2+ APOE+ macrophages are overall closer to EMT hotspots compared to EPI hotspots, there is heterogeneity within each slide and across different slides.”

A summary of the findings from analysing the features around each individual hotspot is addressed in Results, lines 306-321:

“We next sought to interrogate spatial relationships at a more granular level, and analysed the association of EMT hotspots with other immune and stromal areas within the same slide and across the different patient samples **(Fig. 4a).** Whilst the cells that had the strongest relationship with EMT hotspots when averaged over the slides, such as SIGLEC+ and LAM2+ APOE+ macrophages and CAFs, displayed the most consistent trends across the slides, it was evident that these relationships were still heterogenous. For example, in slide 4, whilst seven EMT hotspots were closer to LAM2+ APOE+ macrophages than the median EPI hotspots, two were not **(Fig. 4a).** This is further illustrated through visual inspection of the individual EMT hotspots and comparison with the LAM2+ APOE+ macrophage hotspots, enabled through SpottedPy functions **(Fig. 4b).** As expected, stronger associations with myCAFs and macrophage subtypes were pervasive in most tumours and hotspots **(Fig. 4a).** T-cells demonstrated noticeable heterogeneity across patients, but clustered together, reinforcing the idea that these cells share common response patterns. The EMT hotspots that were closer to T-cells were more likely to be enriched in exhaustion markers **(Fig. 4a, right panel),** suggestive of chronic immune activation. We also show that EMT hotspots show a consistent trend of displaying higher suppressive scores than EPI hotspots **(Fig. 4a).** Overall, the inter-patient heterogeneity seemed to supersede the intra-patient heterogeneity.“

Other comments are as follows:

1.      Is there any inconsistency in EMT signature score distribution among different samples? The author only shows individual slides in each figure.

The question of inter-patient heterogeneity is an excellent one and one which we have not addressed sufficiently. We now include an overview of EMT hotspots identified across all the slides analysed in Supplementary Figures 1c-d, where a certain degree of heterogeneity in the number and spatial distribution can be observed. We comment on these aspects in the Results section, lines 198-209:

“A certain degree of heterogeneity in the number and spatial distribution of EMT hotspots could be observed across the cohort **(Supplementary Fig. 1c).** Notably, Slides 3, 7 and 10 exhibited the greatest dispersion, which was independent of cancer subtype (TNBC and ER+ HER2+). This dispersion did not influence overall EMT relationships with other cell types **(Supplementary Fig. 2a)**. In contrast, Slides 1 and 5 showed minimal dispersion, also without affecting EMT relationships with other cell types. The distributions of individual EMT and EPI signature scores per slide are highlighted in **Supplementary Fig. 1c-d**. The EPI signature typically followed a normal distribution across spatial tumour spots, whereas the EMT signature exhibited a positive skew, in line with this state being expected to be rarer within the primary tumours. Slides 0, 1, and 9 display more pronounced skewness for the EPI signature, suggesting a more advanced stage of EMT transformation within these tumours, which was not an effect of the cancer subtype as these slides present different breast cancer pathologies.”

We would also like to highlight that we now more extensively explore intra- and interpatient heterogeneity in EMT hotspots and their interplay with the tumour microenvironment in a new section entitled **“EMT hotspots display intra- and inter-patient heterogeneity”** and linked **Figure 4** (addressing your point 7 below).

2.      Hotspots of EMT, hypoxia, and angiogenesis may be co-located in the same spots. However, the authors show in the rightmost panel of Figure 2A that spots with these three characteristics are mutually exclusive.

That is a good point. We have adjusted the plot to display the spots with multiple signatures (see Figure 2a).

3.      The authors showed proliferation hotspots are closer to EMT coldspots, but the authors didn't show the distribution of proliferation hotspots.

We have added a panel in Figure 2a to show the proliferative hotspots (2^nd^ panel).

4.      What do the colours in Figure 2b and e mean?

We have now clarified in the figure caption that these colours reflect the magnitude of distances between hotspots. Thus, smaller size and darker colour of the dots indicate closer proximity to hotspot.

5.      How did the authors calculate the tumour perimeter? Cottrazm (10.1038/s41467-023-36560-7) developed by Xun et al. can delineate the location of tumour boundaries. The authors can compare with their method to confirm whether similar results can be obtained.

We have clarified the calculation in the methods section, lines 810-821:

“**Tumour perimeter calculation**

Any spot was considered part of the tumour perimeter if it had more than one neighbouring spots (nodes in the graph) that were not classified as tumour spots. A spot $s$ $\epsilon$ $S$ is considered part of the tumour perimeter, $P,$ if:

Where $S$ denotes the set of all spots, $T$ denotes the set of tumour spots $N\left( s \right)$ represents the neighbouring spots of spot $s$. This approach helped us to delineate the boundary of the tumour accurately by focusing on the transitional area where tumour and non-tumour spots meet (called using *sp.calculate_tumour_perimeter*). To quantify the number of tumour hotspots, we calculated the number of connected components within the graph that were labelled as hotspots. This calculation was crucial for understanding the distribution and clustering of tumour cells. “

We additionally compared the perimeter to Cottrazm’s identification of the perimeter by comparing the results on the same slide, and confirm similar results (lines 225-227):

“We conducted a visual benchmark of our tumour perimeter calculation against the Cottrazm method(40), confirming that our algorithm accurately captures similar perimeter trends (**Supplementary Fig. 2b**). “

See our Supplementary Fig. 2b against Figure 2a in Xun et al:

Supplementary Fig 2b (orange denotes the perimeter):

Figure 2a in Xun et al, Nat Commun 2023 (blue denotes the perimeter):

6.      In Figure 3e and f, macrophages ranked in the top 6 and top 2 respectively in the ranking close to EMT hotspots. What causes this difference?

The differences between the two panels stem from the reference we are using when we perform the calculation. In panel e (now d in the updated manuscript) we highlight the distances from any EMT hotspot to any other immune or stromal hotspot. In panel f (now e) we highlight the relative proximity to either an EMT hotspot or an EMT coldspot. This highlights the importance of our differential spatial relationship calculation: absolute distances only offer limited information, and if we want to assess the significance of certain relationships then we need to compare areas where we see an enriched EMT signature to those where we don’t see one. In this way, panel f (now e) sheds light on which of the immune and stromal regions may be significantly closer to the EMT hotspots, and thus more biologically meaningful. We have emphasised this now in the Results in lines 251-257:

“Quantifying hotspot distances using SpottedPy allowed us to confirm that tumour EMT hotspots were indeed closer to myCAF hotspots (**Fig. 3d**). The relationship is particularly highlighted when we look at the cellular niches that are significantly closer to EMT hotspots compared to EPI hotspots, revealing a predominance of various CAF subtypes. This is well in line with existing studies, as myCAFs have been shown to produce TGF-β, which is a well-known EMT trigger(48). They have also been linked to ECM deposition and suppression of antitumor immunity(49)^,^(50)^,^(51)^,^(52).“

7.   Although macrophage and myCAF are relatively close to EMT hotspots, they are close to EMT hotspots at different spatial locations in Fig. 3. Do different EMT hotspots have different characteristics?

This is an interesting point and we have added functionality in SpottedPy to interrogate different EMT hotspots. This is addressed in lines 306-326 in the new section entitled **“EMT hotspots display intra- and inter-patient heterogeneity”**:

“We next sought to interrogate spatial relationships at a more granular level, and analysed the association of EMT hotspots with other immune and stromal areas within the same slide and across the different patient samples **(Fig. 4a).** Whilst the cells that had the strongest relationship with EMT hotspots when averaged over the slides, such as SIGLEC+ and LAM2+ APOE+ macrophages and CAFs, displayed the most consistent trends across the slides, it was evident that these relationships were still heterogenous. For example, in slide 4, whilst seven EMT hotspots were closer to LAM2+ APOE+ macrophages than the median EPI hotspots, two were not **(Fig. 4a).** This is further illustrated through visual inspection of the individual EMT hotspots and comparison with the LAM2+ APOE+ macrophage hotspots, enabled through SpottedPy functions **(Fig. 4b).** As expected, stronger associations with myCAFs and macrophage subtypes were pervasive in most tumours and hotspots **(Fig. 4a).** T-cells demonstrated noticeable heterogeneity across patients, but clustered together, reinforcing the idea that these cells share common response patterns. The EMT hotspots that were closer to T-cells were more likely to be enriched in exhaustion markers **(Fig. 4a, right panel),** suggestive of chronic immune activation. We also show that EMT hotspots show a consistent trend of displaying higher suppressive scores than EPI hotspots **(Fig. 4a).** Overall, the inter-patient heterogeneity seemed to supersede the intra-patient heterogeneity.

SpottedPy offers functionality to inspect the distance distributions for hotspots within a slide **(Fig. 4c),** and across each slide **(Fig. 4d).** Visualising these distributions highlights that whilst LAM2+ APOE+ macrophages are overall closer to EMT hotspots compared to EPI hotspots, there is heterogeneity within each slide and across different slides.“

8.      Three methods were used to compare the association of hotspot in EMT with other cell features, and whether the authors can compare the accuracy of these methods and whether these methods are applicable in other cancer types.

We have simplified the description of these methods in Figure 1 and emphasised the main method (shortest path to hotspot) which is the default one in SpottedPy as we consider it to be the most sensible approach for interrogating spatial relationships. Supplementary Figure 3e highlights the disadvantage of the centroid approach that we have decided not to include as a method. Identifying a suitable metric to compare these methods is not entirely straightforward, as they all provide different statistics to measure distances between areas within a tissue and they are all in principle applicable – it is more about the interpretation of these distances and whether that is biologically meaningful or not (Supplementary Fig 3e illustrates this further). To demonstrate the shortest path approach accurately captures distances between hotspots within the tissue, we have now simulated a hypoxia hotspot moving away from a mesenchymal hotspot, see Supplementary Figure 1e and lines 211-215 in the Results section:

“To check that that SpottedPy accurately measures hotspot distance using the shortest path approach, we simulated a hypoxia hotspot moving away from a mesenchymal hotspot of interest (**Supplementary Fig. 1e**). As the hypoxia hotspot moves farther away from the mesenchymal hotspot the calculated distance between the hotspots increases, as expected.”

Regarding the question on whether these are applicable to other cancers, all of these distance metrics are applicable to any spatial transcriptomics dataset. To showcase this, we have expanded our analyses and applied the hotspot method to three other cancer types. We present the results of this analysis in a new section entitled **“Spatial EMT relationships in other cancer types”** (lines 372-394) and the linked Supplementary Figure 4:

“We further investigated whether the relationships observed for EMT hotspots in breast cancer were consistent across other cancer types. We assessed these relationships in publicly available datasets from basal cell carcinomas (BCC)(67), pancreatic ductal adenocarcinomas (PDAC)(68) and colorectal cancers (CRC)(69).

Within the BCC slides, angiogenetic and hypoxic hotspots were closer to EMT hotspots **(Supplementary Fig. 4a-b).** Interestingly, proliferative hotspots were also closer to EMT hotspots, suggesting an alternative relationship compared to breast cancer. POSTN+ fibroblasts were closer to EMT hotspots, whilst there were no significant spatial relationships with T cells or NK cells, paralleling the findings in breast cancer.

We next assessed the relationships within one available PDAC slide **(Supplementary Fig. 4c).** Both angiogenesis and fibroblasts displayed a spatial relationship like that observed with EMT hotspots in breast cancer. In contrast, immune cells were located nearer to EMT coldspots, and there was no significant association between EMT hotspots and hypoxic environments, diverging from the patterns observed in breast cancer.

In CRC, myofibroblasts, angiogenesis, and hypoxia showed comparable spatial relationships to those seen in breast cancer **(Supplementary Fig. 4d-e).** Notably, regulatory T-cells, T-helper cells, and NK cells were significantly closer to EMT hotspots, potentially indicating enhanced immune recognition around EMT hotspots compared to other cancer types.

While limited in breadth and cell type resolution, these analyses suggest that the interplay between tumour cells undergoing EMT and other immune and stromal cells within the TME is likely to be tissue specific, and future work should explore this in more detail.”

We further comment on the limitations of this analysis in the Discussion section in lines 593-600:

“When investigating to what extent these spatial EMT relationships are maintained or differ across cancer types other than breast cancer, we were limited by the availability and size of such datasets, as well as the differences in cell composition between tissues. Ultimately, any uncovered differences are likely attributed to the unique TME and genetic basis of each cancer type, and in the future a more in-depth analysis in larger datasets once these become more widely available will shed light on the heterogeneity of these relationships. Additionally, extending these analyses to single cell-resolved spatial datasets and incorporating ligand-receptor signalling information into the evaluation of spatial effects on cell populations will increase the confidence in the identified relationships.“

9.      The authors only showed the distance of different cell types with EMT hotspots and coldspots, do different EMT characteristics of tumours affect their interactions with other cell types?

Due to the limited size of the spatial transcriptomics dataset, we are underpowered to assess whether any EMT relationships are breast cancer subtype-specific rather than a universally observed phenotype. However, we do highlight associations per patient sample in Supplementary Fig. 2a to emphasise that a degree of heterogeneity is to be expected. Cancer subtype-specific trends may become more apparent with a larger sample size and future analyses should investigate this.

This question may also link to the Reviewer’s previous excellent point in Q7 above, of whether local microenvironmental niches may emerge that show different relationships with EMT. To enable this, we have added functions to help users distinguish different hotspots, see Results lines 306-326:

“We next sought to interrogate spatial relationships at a more granular level, and analysed the association of EMT hotspots with other immune and stromal areas within the same slide and across the different patient samples **(Fig. 4a).** Whilst the cells that had the strongest relationship with EMT hotspots when averaged over the slides, such as SIGLEC+ and LAM2+ APOE+ macrophages and CAFs, displayed the most consistent trends across the slides, it was evident that these relationships were still heterogenous. For example, in slide 4, whilst seven EMT hotspots were closer to LAM2+ APOE+ macrophages than the median EPI hotspots, two were not **(Fig. 4a).** This is further illustrated through visual inspection of the individual EMT hotspots and comparison with the LAM2+ APOE+ macrophage hotspots, enabled through SpottedPy functions **(Fig. 4b).** As expected, stronger associations with myCAFs and macrophage subtypes were pervasive in most tumours and hotspots **(Fig. 4a).** T-cells demonstrated noticeable heterogeneity across patients, but clustered together, reinforcing the idea that these cells share common response patterns. The EMT hotspots that were closer to T-cells were more likely to be enriched in exhaustion markers **(Fig. 4a, right panel),** suggestive of chronic immune activation. We also show that EMT hotspots show a consistent trend of displaying higher suppressive scores than EPI hotspots **(Fig. 4a).** Overall, the inter-patient heterogeneity seemed to supersede the intra-patient heterogeneity.

SpottedPy offers functionality to inspect the distance distributions for hotspots within a slide **(Fig. 4c),** and across each slide **(Fig. 4d).** Visualising these distributions highlights that whilst LAM2+ APOE+ macrophages are overall closer to EMT hotspots compared to EPI hotspots, there is heterogeneity within each slide and across different slides.”

10.     Hotspots and coldspots were identified based on Getis-Ord G* statistic. However, it seems that the spatial similarity between hotspot and other immune features in EMT is assessed by distance, and the description of coldspot seems to be less

We have clarified the calculation of hotspots and coldspots in the methods section in lines 737 to 770:

“Hotspots were calculated using The Getis-Ord G* statistic as implemented using the PySAL package(99), using 10 as neighbourhood size parameter by default and a p-value of 0.05, unless otherwise stated.

The Getis-Ord G* equation is defined as follows:

<https://www.codecogs.com/eqnedit.php?latex=G_i%5E*%20%3D%20%5Cfrac%7B%5Csum_%7Bj%3D1%7D%5E%7Bn%7D%20w_%7Bij%7D%20x_j%20-%20%5Cbar%7Bx%7D%20%5Csum_%7Bj%3D1%7D%5E%7Bn%7D%20w_%7Bij%7D%7D%7Bs%20%5Csqrt%7B%5Cfrac%7Bn%20%5Csum_%7Bj%3D1%7D%5E%7Bn%7D%20w_%7Bij%7D%5E2%20-%20(%5Csum_%7Bj%3D1%7D%5E%7Bn%7D%20w_%7Bij%7D)%5E2%7D%7Bn-1%7D%7D%7D#0>

Where<https://www.codecogs.com/eqnedit.php?latex=w_%7Bij%7D#0> is the spatial weight between location<https://www.codecogs.com/eqnedit.php?latex=i#0> and<https://www.codecogs.com/eqnedit.php?latex=j#0> ,<https://www.codecogs.com/eqnedit.php?latex=%5Cbar%7Bx%7D#0> is the mean of the variable of interest across all locations,<https://www.codecogs.com/eqnedit.php?latex=s#0> is the standard deviation of the variable of interest across all locations and<https://www.codecogs.com/eqnedit.php?latex=n#0> is the total number of locations.

A high positive value at location<https://www.codecogs.com/eqnedit.php?latex=%5Ctextit%7Bi%7D#0> suggests a hotspot for the attribute, while a negative value indicates a coldspot. The significance of<http://www.texrendr.com/?eqn=%5Ctextit%7BG%5E*%7D#0> is determined by comparing the observed<http://www.texrendr.com/?eqn=%5Ctextit%7BG%5E*_%7B%5Ctext%7Bobs%7D%7D%7D#0> to a distribution of<http://www.texrendr.com/?eqn=%5Ctextit%7BG%5E*%7D#0> values generated under the assumption of spatial randomness. This distribution is obtained by permuting the attribute values across locations and recalculating<http://www.texrendr.com/?eqn=%5Ctextit%7BG%5E*%7D#0> for each permutation. The p-value for a hotspot (when<http://www.texrendr.com/?eqn=%5Ctextit%7BG%5E*%7D#0> is positive) or a coldspot (when<http://www.texrendr.com/?eqn=%5Ctextit%7BG%5E*%7D#0> is negative) is then derived from this distribution. This approach provides a non-parametric method to evaluate the significance of spatial clusters, offering a robust measure against potential spatial randomness in the data. The significance of is determined by comparing the observed to a distribution of values generated under the assumption of spatial randomness. This distribution is obtained by permuting the attribute values across locations and recalculating for each permutation. The p-value for a hotspot (when is positive) or a coldspot (when is negative) is then derived from this distribution. This approach provides a method to evaluate the significance of spatial clusters, offering a robust measure against potential spatial randomness in the data. […]

Hotspots and coldspots for EMT states and cell proliferation were calculated after filtering for tumour cells as labelled by STARCH. All other hotspots (deconvolved cell proportion data and angiogenic and hypoxia signatures) were calculated using all the spots within the spatial transcriptomic slide. ”

In the Results section, we have chosen to place more emphasis on the relationships observed with EMT hotspots because those are of particular biological interest, whereas coldspots are simply areas that lack a signature and thus we feel their best use is to provide a contrast and a comparison term for the hotspots.

11.     There are dozens of feature genes for immune suppression and ICB response. What are the author's criteria for selecting these genes?

The immune suppression and ICB response gene signatures employed in the analyses had been reported by Johnson et al and Wu et al. We have now expanded our gene lists to include other signatures for immune suppression from Cui et al and another signature for ICB response from Liu et al to ensure it is less biased towards any single signature. We observe the same trends as previously reported, see Figures 3f-i, 6c-e.

Signatures:

Johnson AM, Bullock BL, Neuwelt AJ, Poczobutt JM, Kaspar RE, Li HY, et al. Cancer Cell-Intrinsic Expression of MHC Class II Regulates the Immune Microenvironment and Response to Anti-PD-1 Therapy in Lung Adenocarcinoma. J Immunol. 2020 Apr 15;204(8):2295–307.

Wu SZ, Al-Eryani G, Roden DL, Junankar S, Harvey K, Andersson A, et al. A single-cell and spatially resolved atlas of human breast cancers. Nat Genet. 2021 Sep;53(9):1334–47.

Liu J, Ling Y, Su N, Li Y, Tian S, Hou B, et al. A novel immune checkpoint-related gene signature for predicting overall survival and immune status in triple-negative breast cancer. Transl Cancer Res. 2022 Jan;11(1):181–92.

Cui C, Xu C, Yang W, Chi Z, Sheng X, Si L, et al. Ratio of the interferon-γ signature to the immunosuppression signature predicts anti-PD-1 therapy response in melanoma. NPJ Genom Med. 2021 Feb 4;6(1):7.

12.     The author needs to elaborate on how to use Spottedby. The methods need to be reorganized to make them clear to the reader.

We thank the reviewer for this suggestion. We have added more detail in the Methods section on how to run SpottedPy, including the function names that should be called at the relevant part within the methods (please see sections highlighted in yellow). We would also like to emphasise that we have included detailed tutorials on how to use SpottedPy on GitHub, see [https://github.com/secrierlab/SpottedPy/blob/main/spottedPy/tutorials/[spottedPy_multiple_slides.ipynb](https://github.com/secrierlab/SpottedPy/blob/main/spottedPy/tutorials/spottedPy_multiple_slides.ipynb)](https://github.com/secrierlab/SpottedPy/blob/main/spottedPy/tutorials/spottedPy_tutorial_one.ipynb) and [https://github.com/secrierlab/SpottedPy/blob/main/spottedPy/tutorials/spottedpy_tutorial_sample_dataset.ipynb.](https://github.com/secrierlab/SpottedPy/blob/main/spottedPy/tutorials/spottedpy_tutorial_sample_dataset.ipynb)
We now point to these tutorials also in the Methods in lines 624-632:

**“The SpottedPy package**

SpottedPy package is compatible with Python 3.9 and depends on scanpy, libpysal, esda packages. The key functions are outlined in the relevant sections below.

- GitHub: <https://github.com/secrierlab/SpottedPy>
- Tutorials: spottedPy_multiple_slides.ipynb (this tutorial walks through using SpottedPy with multiple spatial slides, highly recommended for downstream statistical analysis). spottedpy_tutorial_sample_dataset.ipynb tutorial walks through using SpottedPy with a single slide.
- Sample data: <https://zenodo.org/records/10392317>”

We have also expanded our GitHub repository documentation to include more details on the functions offered by the package, please see: <https://github.com/secrierlab/SpottedPy>

**Reviewer #2**

Withnell et al. developed SpottedPy to identify tumor hotspots in spatial transcriptomics slides and explore their interaction with the tumor microenvironment (TME) at different scales. Using breast cancer as a model, the authors investigate epithelial-mesenchymal plasticity and emphasize locally stable niches linked to angiogenic and hypoxic regions. Additionally, the authors modified various parameters, such as neighborhood size and distance metrics, to assess the robustness and efficacy of SpottedPy. to identify tumour hotspots in spatial transcriptomics slides and explore their interaction with the tumour microenvironment (TME) at different scales. Using breast cancer as a model, the authors investigate epithelial-mesenchymal plasticity and emphasize locally stable niches linked to angiogenic and hypoxic regions. Additionally, the authors modified various parameters, such as neighbourhood size and distance metrics, to assess the robustness and efficacy of SpottedPy. However, some conclusions in the paper still necessitate further clarification.

1.All conclusions are based on breast cancer, yet the paper's title does not reflect this. The authors should include more types of cancer to determine whether the conclusions regarding EMT are applicable across different cancer types

We have changed the title to reflect the breast cancer focus to: “SpottedPy quantifies relationships between spatial transcriptomic hotspots and uncovers new environmental cues of epithelial-mesesenchymal plasticity in breast cancer”. Furthermore, we have run additional studies on other cancer types and include the results in an entirely new section entitled **“Spatial EMT relationships in other cancer types”** in the revised version of the manuscript. Lines 372-394 describe this:

“We further investigated whether the relationships observed for EMT hotspots in breast cancer were consistent across other cancer types. We assessed these relationships in publicly available datasets from basal cell carcinomas (BCC)(67), pancreatic ductal adenocarcinomas (PDAC)(68) and colorectal cancers (CRC)(69).

Within the BCC slides, angiogenetic and hypoxic hotspots were closer to EMT hotspots (Supplementary Fig. 4a-b). Interestingly, proliferative hotspots were also closer to EMT hotspots, suggesting an alternative relationship compared to breast cancer. POSTN+ fibroblasts were closer to EMT hotspots, whilst there were no significant spatial relationships with T cells or NK cells, paralleling the findings in breast cancer.

We next assessed the relationships within one available PDAC slide (Supplementary Fig. 4c). Both angiogenesis and fibroblasts displayed a spatial relationship like that observed with EMT hotspots in breast cancer. In contrast, immune cells were located nearer to EMT coldspots, and there was no significant association between EMT hotspots and hypoxic environments, diverging from the patterns observed in breast cancer.

In CRC, myofibroblasts, angiogenesis, and hypoxia showed comparable spatial relationships to those seen in breast cancer (Supplementary Fig. 4d-e). Notably, regulatory T-cells, T-helper cells, and NK cells were significantly closer to EMT hotspots, potentially indicating enhanced immune recognition around EMT hotspots compared to other cancer types.

While limited in breadth and cell type resolution, these analyses suggest that the interplay between tumour cells undergoing EMT and other immune and stromal cells within the TME is likely to be tissue specific, and future work should explore this in more detail.”

We further comment on this in the discussion in lines 593-600:

“When investigating to what extent these spatial EMT relationships are maintained or differ across cancer types other than breast cancer, we were limited by the availability and size of such datasets, as well as the differences in cell composition between tissues. Ultimately, any uncovered differences are likely attributed to the unique TME and genetic basis of each cancer type, and in the future a more in-depth analysis in larger datasets once these become more widely available will shed light on the heterogeneity of these relationships. Additionally, extending these analyses to single cell-resolved spatial datasets and incorporating ligand-receptor signalling information into the evaluation of spatial effects on cell populations will increase the confidence in the identified relationships.”

2.Which spots were used to calculate different types of hotspots? For example, in Figure 2A, the authors initially used STRACH to identify tumor spots and then applied Getis-Ord G* statistics to determine EMT hotspots or coldspots among them. However, were all spots used for hypoxic and angiogenic spots?

This is a useful point that we have now clarified in the Methods section in lines 768 to 770:

“Hotspots and coldspots for EMT states and cell proliferation were calculated after filtering for tumour cells as labelled by STARCH. All other hotspots (deconvolved cell proportion data and angiogenic and hypoxia signatures) were calculated using all the spots within the spatial transcriptomic slide. […]

After calculating the hotspots and coldspots, we then assessed the distances from hotspots of interest (EPI and EMT) to other cells types and signature hotspots and coldspots.”

3.In Fig. 2A (right panel) and Fig. 3D (left panel), the authors overlaid different types of hotspots/coldspots on the same slide. An issue arises when different spot types overlap—how to assign names to these spots. For instance, there might be overlap between hotspot points for myCAF and hypoxic spots. How should this be addressed during the calculation process?

This is a pertinent point that was also raised by Reviewer #1. We have now adjusted the plot to represent these relationships more accurately. We now display the spots with multiple signatures, (see Figure 2a). We have removed the previous 3D panel for the sake of space and panel balance in Figure 3.

For the purpose of hotspot calculation, the overlap is not an issue as each spot can be assigned to multiple hotspots at the same time and the distance calculation can accurately represent this.

4.The author did not clearly explain how to calculate the tumour perimeter in the methods section. For example, some spots on the left side of Figure 2D are labelled as tumour perimeter, yet they are not surrounded by any tumour spots, which is evidently unreasonable. Therefore, the author should provide a rigorous mathematical formula for definition.

We thank the Reviewer for raising this important point, which is similar to a comment from Reviewer #1. We have improved the perimeter determination method so that it is more accurate (we consider a spot in perimeter if more than 1 spot surrounding a tumour spot is not a tumour spot, whereas previously this was set to 2) and compared the perimeter to Cottrazm’s (Xun et al, Nat Commun 2023) identification of the perimeter by comparing the results on the same slide (lines 225-227):

“We conducted a visual benchmark of our tumour perimeter calculation against the Cottrazm method(40), confirming that our algorithm accurately captures similar perimeter trends (**Supplementary Fig. 2b**).”

See our Supplementary Fig. 2b against Figure 2a in Xun et al:

Supplementary Fig 2b (orange denotes the perimeter):

Figure 2a in Xun et al, Nat Commun 2023 (blue denotes the perimeter):

We have clarified the calculation in the Methods section, line 812-821:

“Any spot was considered part of the tumour perimeter if it had more than one neighbouring spots (nodes in the graph) that were not classified as tumour spots. A spot $s$ $\epsilon$ $S$ is considered part of the tumour perimeter, $P,$ if:

Where $S$ denotes the set of all spots, $T$ denotes the set of tumour spots $N\left( s \right)$ represents the neighbouring spots of spot $s$. This approach helped us to delineate the boundary of the tumour accurately by focusing on the transitional area where tumour and non-tumour spots meet (called using *sp.calculate_tumour_perimeter*). To quantify the number of tumour hotspots, we calculated the number of connected components within the graph that were labelled as hotspots. This calculation was crucial for understanding the distribution and clustering of tumour cells.”

5.To some extent, using the shortest distance may not be suitable for calculating the similarity between two sets of spots. For instance, when EMT hotspots and proliferative hotspots share at least one common spot, their distance is zero. However, centroid-to-centroid distance also has significant limitations. For example, when EMT hotspots form irregular shapes or consist of multiple clusters distributed across different areas, the centroid approach may lead to erroneous conclusions.

We thank the Reviewer for this useful comment. As we calculate all the minimum distances for each spot within each hotspot and then calculate a summary statistic, it is not a problem when two hotspots share the same spot. Additionally, this mitigates issues that arise when EMT hotspots form irregular shapes or consist of multiple clusters distributed across different areas. We have adjusted lines 775-788 in the methods section to highlight this:

“We used the ***shortest path*** approach to calculate distances between hotspots as follows:

- Let<https://www.codecogs.com/eqnedit.php?latex=H#0> represent the set of coordinates of spots in the hypoxia hotspot.
- Let<https://www.codecogs.com/eqnedit.php?latex=M#0> represent the set of coordinates of spots in the mesenchymal tumour hotspot.
- Let<https://www.codecogs.com/eqnedit.php?latex=E#0> represent the set of coordinates of spots in the epithelial tumour hotspot.

For a spot<https://www.codecogs.com/eqnedit.php?latex=m#0> in<https://www.codecogs.com/eqnedit.php?latex=M#0> and a spot<https://www.codecogs.com/eqnedit.php?latex=e#0> in<https://www.codecogs.com/eqnedit.php?latex=E#0>, the shortest path to any point<https://www.codecogs.com/eqnedit.php?latex=h#0> in<https://www.codecogs.com/eqnedit.php?latex=H#0> was determined:

Where $d\left( m,h \right)$ represents the Euclidean distances from a spot $m$ in $M$. After obtaining the minimum distances for each spot in $M$ and $E$ we calculated the median (with the additional functionality to choose min or mean) to provide a summary statistic that reflects the geneal proximity of each hotspot ($M$ and $E$) to $H$.”

6.Line 283-284: "We found that NK cells were the only cells consistently adjacent to EMT coldspots at various hotspot sizes." Based on Figure 2SA, it appears that this conclusion cannot be drawn.

The reviewer is right to point out that there is inter-patient variability of all observed relationships. We have now removed this line.

7.Sensitivity analysis should be emphasized. From Figure 4, it is evident that EMT hotspots vary significantly with different neighbour sizes, indicating instability. This raises questions about the meaningfulness of the hotspot definition.

We thank the Reviewer for raising this key point. We have run further analysis to assess the reliability of hotspots found. We have addressed this in the Results, lines 348-356 (see also Figures 5e-f):

“To evaluate the stability of the spatial relationships, we introduced gaussian noise and spot reshuffling into the dataset and examined the persistence of these relationships (see Methods). This approach demonstrated that the method is robust to low levels of noise, but also effectively discriminates between biologically meaningful signals from those arising from random spatial distributions **(Fig. 5e-f).** While random noise simulated through spot reshuffling can mimic some aspects of structured data (**Supplementary Fig. 3c**), the hotspots are significantly smaller than those that are biologically relevant (**Supplementary Fig. 3d**). Crucially, the loss of specific associations among particular cell types when noise is introduced **(Fig 5e-f)** contributes to the reduction of false positives even if hotspots are identified(66). “

A hotspot refers to a region within a tissue sample where signatures occur at significantly higher intensities compared to surrounding areas (the neighbourhood). The appropriate size for a neighbourhood is fundamentally a biological question, dependent on the spatial relationships and scales relevant to the biological phenomena being studied. This variability underscores the importance of our approach, where we systematically evaluate how spatial relationships shift with changes in neighbourhood sizes to robustly define hotspots. The instability is therefore an inherent property of a spatial signal, which we have tried to emphasise in Figure 5 and Supplementary Figure 3.

8.All subsequent results depend on the accuracy of tumour spot identification. I believe that in sensitivity analysis, the authors should consider the impact of SCRACH on different parameters (e.g. setting K=?? clones), or rely on experts to assess the accuracy of tumour spot identification. This would make downstream analysis more practically meaningful.

We agree with the Reviewer that this is an important point. Altering value of K did not affect tumour cell identification by visual inspection, but changed how many tumour clones were identified (which was information returned from STARCH that we did not require for downstream analysis). This is highlighted in the Methods section in lines 671-672:

“Changing the value of K alters the number of identified tumour clones, but the number of cells labelled as tumour cells remains the same.”

We compared the accuracy of tumour spot identification to expert annotation, highlighted in Supplementary Fig. 1a-b. This is highlighted in lines 177-179:

“We validated the STARCH results by comparing to publicly available pathologist annotated slides available (Supplementary Fig. 1a-b).”

9.What is the meaning of "#Rings around spot" in Fig. 5b? Based on Fig. 1A, the first ring should include 6 spots, why did the author specifically choose numbers like 1, 2, 3, 4, 5, 10, 20, 50 and 100?

We have clarified this in the Supplementary Figure 5 legend:

“Each ring represents an incremental distance from the central spot and encompasses the surrounding spatial transcriptomic spots.”

We have additionally clarified this in the Methods section in lines 722-728:

“The first ring consists of 6 spots, and the second ring includes 18 spots (combined from the 1st and 2nd rings). Subsequent rings follow this pattern. The number of rings selected for sensitivity analysis reflects a balance between spatial coverage and resolution. Using a smaller number of rings (e.g., 1, 2, 3) allows the analysis to focus on the immediate microenvironment around the central spot, providing high resolution. As more rings are added, the spatial coverage increases, capturing broader interactions but potentially diluting local-specific signals.”

10.What's the meaning of "number of components" in Fig.4d

This refers to the method of calculating the number of hotspots. We have adjusted this in the figure (now Figure 5d) to make it clearer to the reader.

11.What's the meaning of "correlation change" in Fig.5c.

This referred to the change in Pearson correlation coefficient with increasing ring size. This summarised the results in the previous Fig. 5b (now Supplementary Fig. 5b). We have removed this figure panel in the updated manuscript as it was redundant with the current Supplementary Fig. 5b.

**Second round of review**

**Reviewer 1**

The authors highly improved the manuscript and addressed most of my comments. The minor comments as follows:

1. The reviewer’s previous comments that about do the different EMT hotspots associate with TME. The authors performed the intra- and inter-patient heterogeneity of EMT hotspots. Based on their additional results in Fig. 4 and the authors defined the tumor, tumor perimeter, and TME region. How the location of EMT hotspots in these three regions?
2. The Figure 4b showed hotspots of slide 4 while the Fig 4c showed the distance to o LAM2+ APOE+ macrophage of slide 4. Keep the consistent example will help the readers capture the results.
3. The Fig. 4b and Fig. 5c used the same slide, the EMT hotspots look like in the different pattern.

**Reviewer 2**

While the revised manuscript has addressed most of the concerns, some questions still require clarification.

1. The author still hasn't clearly defined how to distinguish between different types of hot spots. Regarding my previous 2nd Question, the author responds, "Hotspots and coldspots for EMT states and cell proliferation were calculated after filtering for tumor cells as labeled by STARCH." However, in Figure 2a, the number of spots highlighted as high EMT does not correspond to the number of tumor spots. Despite the comparison with the Cottrazm method, the author's conclusions heavily rely on identifying tumor spots. Any inaccuracies in this identification could lead to erroneous conclusions.

2. When identifying hotspots, the author used the default neighborhood size of 10 (PySal). However, it's clear from Figure 5a that the conclusions are not sufficiently stable. How should users choose this parameter when using this method?

3. In Figures 2b and 2e, dot plots do not effectively represent the distribution distances. Would it be more appropriate to use violin plots or box plots?

4. Many isolated points are identified as perimeters (within tumor spots). How should these isolated points be interpreted?

**Authors Response**

**Point-by-point responses to the reviewers’ comments:**

**SUMMARY OF MANUSCRIPT CHANGES – Withnell and Secrier**

We thank the Reviewers for their additional suggestions, which have been fundamental to clarifying some aspects of our work. As a result, we have made the following modifications to the manuscript:

1. We have improved our method to identify the tumour perimeter by filtering out isolated points which were marked as pertaining to the perimeter. We have updated the corresponding tumour perimeter proximity calculations in Figure 2 and Supplementary Figure 2, and also display distances between individual EMT hotspots and the tumour perimeter in Figure 4a.
2. We further clarify the calculation of EMT and EPI hotspots, and the fact that they are only captured within the tumour cells by design. We discuss linked limitations of this approach.
3. We improved the visualisation in Figures 2b and 2e by changing the dot plots to box plots.
4. We clarified how the neighbourhood parameter should be set for a specific analysis in the Methods and GitHub repository.

A point-by-point description of the changes implemented to address the Reviewers’ comments is included below. The resubmitted manuscript contains tracked changes, with amended Figures 2, 4, 5 and Supplementary Figure 2.

**Reviewer #1:**

The authors highly improved the manuscript and addressed most of my comments. The minor comments as follows:

1. The reviewer’s previous comments that about do the different EMT hotspots associate with TME. The authors performed the intra- and inter-patient heterogeneity of EMT hotspots. Based on their additional results in Fig. 4 and the authors defined the tumor, tumor perimeter, and TME region. How are EMT hotspots located within the tumor, peritumoral, and TME regions?

We thank the Reviewer for raising this point. We have now investigated the proximity of individual EMT hotspots to the tumour perimeter, which helps us describe how the these hotspots are located within the tumour and peritumorally. We observe a consistent trend of EMT hotspots being closer to the tumour perimeter, as would be expected. This has now been added in Figure 4a and commented on in lines 330-332:

“Additionally, nearly all EMT hotspots were closer to the tumour perimeter (**Fig. 4a**), corroborating the overall trends reported in **Fig. 2e-f**.”

Due to the nature of how we calculate EMT hotspots by first filtering for tumour spots only, and then finding EMT hotspots within these tumour spots, we would not find any EMT hotspots within the TME regions **by design**. While we acknowledge that several TME populations present mesenchymal features, e.g. CAFs, we specifically aimed to characterise EMT within tumour cells and not in other immune and stromal cells in our study, hence the filtering for tumour cells only to ensure this signal is tumour-cell specific and not confounded by other factors. Distances between EMT hotspots and other cell populations in the TME are shown in Figure 4a.

2. The Figure 4b showed hotspots of slide 4 while the Fig 4c showed the distance to o LAM2+ APOE+ macrophage of slide 4. Keep the consistent example will help the readers capture the results.

We thank the Reviewer for pointing out that in Figure 4b we show hotspots from slide 4, whereas Figure 4c shows hotspots distances within slide 5. We have now updated Figure 4c to increase consistency as suggested, and present now the distance measurements within slide 4 in line with the slide 4 visualization provided in Figure 4b.

3. The Fig. 4b and Fig. 5c used the same slide, the EMT hotspots look like in the different pattern.

We thank the Reviewer for noticing this discrepancy between Figure 4b and (we assume the Reviewer meant) Figure 5a. Figure 5a had the previous EMT signature score and had not been updated with the new deconvolution results. This has now been updated accordingly, please see the new Figure 5a.

**Reviewer #2**

While the revised manuscript has addressed most of the concerns, some questions still require clarification.

1. The author still hasn't clearly defined how to distinguish between different types of hot spots. Regarding my previous 2nd Question, the author responds, "Hotspots and coldspots for EMT states and cell proliferation were calculated after filtering for tumor cells as labeled by STARCH." However, in Figure 2a, the number of spots highlighted as high EMT does not correspond to the number of tumor spots. Despite the comparison with the Cottrazm method, the author's conclusions heavily rely on identifying tumor spots. Any inaccuracies in this identification could lead to erroneous conclusions.

EMT hotspots are the regions with a high proportion of mesenchymal tumour cells within the tumour-labelled spots. Therefore, they should only include a subset of tumour spots, hence why their number does not correspond to the number of tumour spots (it’s only a subset). We then calculate the EPI hotspots as regions with a high fraction of epithelial tumour cells, also only within the tumour-labelled spots. We hope that the new addition of the EPI hotspot panel in Figure 2a helps to illustrate this. We have also removed the EMT signature gradient panel, as perhaps that was causing confusion.

We have further amended the Methods section (lines 686-687) to clarify how EMT and EPI signatures are scored within the tumour cells:

“We scored the scRNA-seq cancer epithelial cells with EPI and EMT signatures(36,37), and used Gaussian mixture modelling to assign the cells to EPI and EMT clusters.“

When investigating EMT, we specifically aimed to look at this process only within tumour cells. While we acknowledge that several TME populations present mesenchymal features, e.g. CAFs, that is beyond the scope of the study, hence the filtering for tumour cells only to ensure this signal is tumour-cell specific and not confounded by other factors. When it comes to other processes such as hypoxia or angiogenesis, as they are more broadly manifested within the tissue and the tumour microenvironment, we did not perform any filtering for tumour cells.

We have added the following lines in line 808-814 to help further clarify this:

“Hotspots and coldspots for EMT states and cell proliferation were calculated after filtering for tumour cells as labelled by STARCH, as we aimed to specifically capture these processes within the tumour cells themselves. EMT hotspots are the regions with a high proportion of mesenchymal tumour cells within the tumour-labelled spots. Therefore, they only include a subset of tumour spots. Similarly, cell proliferation hotspots are regions with high fractions of proliferating tumour cells. All other hotspots (deconvolved cell proportion data and angiogenic and hypoxia signatures) were calculated using all the spots within the spatial transcriptomic slide. ”

The coldspots, in contrast, are regions where a specific process/signature is absent, and they serve as a reference to compare against the regions with an enrichment of the signature (EMT, hypoxia etc) or cell type. We have added some clarification around how coldspots are calculated in lines 797-802:

“Coldspots are automatically created when *sp.create_hotspots* is called, and hotspots are labelled in the *anndata* object by appending *“_hot”,* and coldspots by appending *“_cold”* to the original column name. When an appropriate contrasting signature is available for comparison e.g. EPI compared to EMT we do not need to use the coldspots for comparison.”

We agree with the Reviewer that it is important to ensure that tumour spots are identified as accurately as possible. We have taken steps to ensure this to the best of our ability, in line with the gold standard in the field. Specifically, to identify the tumour spots we run STARCH, which infers copy number alterations from spatial transcriptomics data. This is arguably the most reliable way we have presently for inferring spots where genomic changes are present, suggesting the cells within those spots must be tumour cells as other cells in the environment are expected to have a normal, diploid genome. Furthermore, we have compared the accuracy of tumour spot identification to expert annotation, highlighted in Supplementary Fig. 1a-b. This is explained in lines 184-186:

“We validated the STARCH results by comparing to publicly available pathologist annotated slides available (Supplementary Fig. 1a-b).”

Additionally, the STARCH method has been validated within the original paper with pathologist annotations and inferCNV^1^.

In all these analyses, we have followed best practices in the field which are widely accepted in publications. However, we acknowledge that further validation of tumour regions, e.g. by staining with specific markers, would be beneficial for users who wish to employ our tools in their experiments and have added a sentence in the Discussion to emphasise this point:

^1^ Elyanow R, Zeira R, Land M, Raphael BJ. STARCH: copy number and clone inference from spatial transcriptomics data. Phys Biol. 2021 Mar 9;18(3):035001. doi: 10.1088/1478-3975/abbe99.

“Here we have leveraged scRNA-seq for deconvolution, alongside detecting copy number aberrations to confirm the presence of tumour cells, which adds a further layer of confidence to the accurate delineation of these cell populations. However, the accuracy of this separation cannot be fully guaranteed and future research investigating this relationship using single-cell resolved spatial transcriptomic data would allow us to confirm this relationship more confidently. We further note that while we have tried to ensure EMT is captured only within the tumour cells themselves by investigating signatures only within the tumour spots identified by STARCH and by using EMT reference datasets from pure tumour populations, further validation of tumour regions, e.g. by staining with specific markers, would be beneficial for users who wish to employ our method in their spatial transcriptomics experiments.”

2. When identifying hotspots, the author used the default neighborhood size of 10 (PySal). However, it's clear from Figure 5a that the conclusions are not sufficiently stable. How should users choose this parameter when using this method?

We have updated and added further description to the readme on GitHub (see <https://github.com/secrierlab/SpottedPy>) and in the Methods section (lines 765-798):

“Hotspots were calculated using The Getis-Ord G* statistic as implemented using the PySAL package(99), using 10 as neighbourhood size parameter by default (number of spot neighbours surrounding the central Visium spot) and a p-value of 0.05, unless otherwise stated. […]

Hotspots can be identified by calling *sp.create_hotspots* function, and specifying in the *filter_columns* parameter what region within the spatial slide to calculate the hotspot from e.g. tumour cells. The *neighourhood*_*parameter* can be altered here (default = 10). We encourage the user to choose the parameter most relevant for their biological question, e.g. whether they are interested in local interactions of the signature, or broader tissue modules. SpottedPy allows the user to perform the sensitivity analysis to observe how the parameters affect downstream analysis. For the 10x Visium platform, we would recommend starting with parameter k=10 as this captures all the spots surrounding the central spot. The variable with the most stable relationships across a range of parameters (and therefore scales) is likely one of most interest for further investigation. However, specific short-range relationships defined locally rather than across scales could also be of interest in certain circumstances depending on the user’s biological questions.”

3. In Figures 2b and 2e, dot plots do not effectively represent the distribution distances. Would it be more appropriate to use violin plots or box plots?

We thank the Reviewer for suggesting this. We have changed the representation to box plots in Figure 2b and Figure 2e.

4. Many isolated points are identified as perimeters (within tumor spots). How should these isolated points be interpreted?

We thank the Reviewer for pointing this out. Isolated perimeter spots are likely artefacts and should be removed from the analysis. We have now addressed this, as described in lines 882-885:

“Additionally, we applied a filtering step to remove isolated perimeter spots. This involved eliminating any identified perimeter spots that had no neighbouring perimeter spots, thereby excluding isolated perimeter spots caused by a single non-tumour labelled spot within the tumour.”

Any isolated spots have been removed and we have updated our calculations and results presented in Figure 2d and Supplementary Figure 2b. There are still isolated groups of perimeter spots, but these delineate the TME infiltrating within the tumour and therefore we assumed a perimeter within the tumour can exist.

**Third round of review**

**Reviewer 1**

The authors have fully addressed my comments. I have not additional comments.

**Reviewer 2**

The authors have addressed my question, and i have no further comments.
